# MRC-SPECT-DF: an MR-Compatible SPECT system with Dual-FOV collimation design for microscopic SPECT imaging

**DOI:** 10.1186/2197-7364-2-S1-A48

**Published:** 2015-05-18

**Authors:** Xiaochun Lai, Chin-tu Chen, Ling-Jian Meng

**Affiliations:** University of Illinois, USA; University of Chicago, USA

We will report the design and performance of a MR-compatible SPECT system that utilizes a dual-FOV aperture design for microscopic SPECT imaging of small animals inside an pre-existing MR scanner. The MRC-SPECT-DF system consists of a full ring of high resolution CdTe detectors and an aperture that consists of dual-FOV aperture system design that has two sets of aperture rings along the axis, one providing a large FOV and the other one allowing an ultrahigh resolution microscopic imaging of a selected target region in the object. The switching between apertures does not require mechanical shifting of either the aperture or the CdTe detector ring. This is critical for integrating the SPECT system with the MR scanner, and minimizing the interference between both sub-system while operation.

